# Kinetic Studies of Newly Patented Aminoalkanol Derivatives with Potential Anticancer Activity as Competitive Inhibitors of Prostate Acid Phosphatase

**DOI:** 10.3390/ijms222111761

**Published:** 2021-10-29

**Authors:** Błażej Grodner, Mariola Napiórkowska, Dariusz Maciej Pisklak

**Affiliations:** 1 Chair and Department of Biochemistry and Pharmacogenomics, Medical University of Warsaw, 1 Banacha Str., 02-097 Warsaw, Poland; 2 Department of Biochemistry, Medical University of Warsaw, 1 Banacha Str., 02-097 Warsaw, Poland; mariola.napiorkowska@wum.edu.pl; 3 Department of Medical, University of Warsaw, 1 Banacha Str., 02-097 Warsaw, Poland; dpisklak@wum.edu.pl

**Keywords:** acid phosphatase inhibitors, anticancer drugs, capillary electrophoresis

## Abstract

Background: Acid phosphatase and its regulation are important objects of biological and clinical research and play an important role in the development and treatment of prostate and bone diseases. The newly patented aminoalkanol (4-[2-hydroxy-3-(propan-2-ylamino)propyl]-1,7-dimethyl-8,9-diphenyl-4-azatricyclo[5.2.1.02,6]dec-8-ene-3,5,10-trione hydrochloride) (I) and (4-[3-(dimethylamino)-2-hydroxypropyl]-1,7-dimethyl-8,9-diphenyl-4-azatricyclo[5.2.1.02,6]dec-8-ene-3,5,10-trione hydrochloride) (II) derivatives have potential anticancer activity, and their influence on enzymatic activity can significantly impact the therapeutic effects of acid phosphatase against many diseases. Therefore, in this study, we investigated the action of compounds (I) and (II) on acid phosphatase. Methods: Capillary electrophoresis was used to evaluate the inhibition of acid phosphatase. Lineweaver–Burk plots were constructed to compare the Km of this enzyme in the presence of inhibitors (I) or (II) with the Km in solutions without these inhibitors. Results: Compound (I) showed a stronger competitive inhibition against acid phosphatase, whereas derivative (II) showed a weaker competitive type of inhibition. The detailed kinetic studies of these compounds showed that their type and strength of inhibition as well as affinity depend on the kind of substituent occurring in the main chemical molecule. Conclusions: This study is of great importance because the disclosed inhibition of acid phosphatase by compounds (I) and (II) raises the question of whether these compounds could have any effect on the treatment possibilities of prostate diseases.

## 1. Introduction

Depending on its origin, acid phosphatase can have different forms. This enzyme can be found in the cellular components of the bone, spleen, kidney, liver, and intestine, as well as in the blood. It also occurs in postpubertal prostatic epithelial cells in exceptionally high concentrations [1,2,3,4,5,6].

The serum levels of prostatic acid phosphatase (PAP) are determined to evaluate the success of the surgical treatment of prostate cancer [4]. In the past, this parameter was also used for the diagnosis of prostate cancer. Acid phosphatase is mostly concentrated in cells and tissues, where it performs a catalytic function. However, in disease conditions, a certain amount of this enzyme tends to leak into the circulation from injured cells and tissues. 

Thus far, many works have focused on acid phosphatase [7,8]. Some have described the properties of acid phosphatase occurring in plants [9,10], while some presented the functioning of the enzyme in the human body [11,12]. The activity of acid phosphatase could be diagnostically valuable as a serological and histological marker of diseases, and also of use in the investigation or treatment of a given disease [13,14,15,16,17,18,19,20].

An important aim of many works is to analyze the possibility of using acid phosphatase activity as a parameter to assess the degree of treatment of prostate cancer-related diseases. Activity of prostatic acid phosphatase increases with the proliferation of malignant tissues [21].

Acid phosphatase is also associated with bone formation processes and may have an important role in the treatment of osteoporosis or osteomalacia. The increasing interest of researchers in the biochemistry of this enzyme is related to its use as a marker of osteoclasts, which are the bone-resorbing cells. Bone demineralization may contribute to an increase in the activity and concentration of acid phosphatase in the serum of affected individuals [22,23].

A pathological increase in bone resorption arises when osteoclasts are stimulated to carry out resorption at an increased rate [24,25,26,27,28,29,30,31]. This upsets the normal balance between bone resorption and bone synthesis [8]. Elevated osteoclast activity is accompanied by an increase in the synthesis, activity, and secretion of acid phosphatase.

The activity of acid phosphatase can be regulated under the influence of many substances [32,33]. Some of these exhibit activation properties [34], whereas others, such as bisphosphonate drugs or molybdate, inhibit the activity of the enzyme [35].

**Figure 1:**
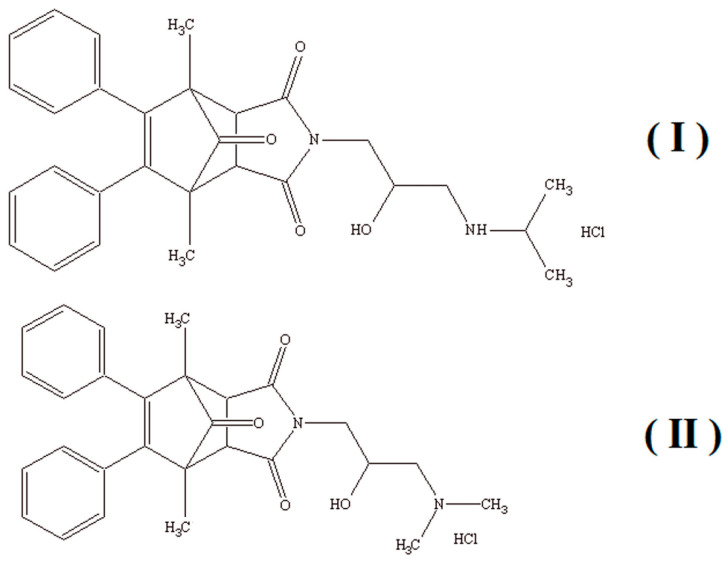
Structures of compounds (**I**) (4-[2-hydroxy-3-(propan-2-ylamino)propyl]-1,7-dimethyl-8,9-diphenyl-4-azatricyclo [5.2.1.0^2,6^] dec-8-ene-3,5,10-trione hydrochloride) and (**II**) (4-[3-(dimethylamino)-2-hydroxypropyl]-1,7-dimethyl-8,9-diphenyl-4-azatricyclo[5.2.1.0^2,6^]dec-8-ene-3,5,10-trione hydrochloride).

Considering the versatility of acid phosphatase and its influence on many biochemical processes in the human body as well as its diagnostic properties, this study aimed to investigate the characteristics and inhibition type of the new derivatives of 1,7-dimethyl-8,9-diphenyl-4-azatricyclo[5.2.1.02,6]dec-8-ene-3,5,10-trione (Figure 1) with a potential anticancer activity, which can act as inhibitors of acid phosphatase. The synthesis, chemical characterization, and anticancer activity of the aforementioned compounds (I) and (II) were described previously in a patent application [36]. 

The present work is a continuation of our studies on aminoalkanol derivatives of dicarboximides [37,38,39,40,41]. In one of our previous studies, we developed a capillary electrophoresis (CE) method for analyzing the inhibition of tissue-non-specific alkaline phosphatase (TNAP) by the aminoalkanol derivatives of dimethyldicarboximide [38]. This paper presents the inhibition studies of PAP, as well as the development and validation of a fast and sensitive CE method for determining the derivatives (I) and (II).

## 2. Results

Compounds (I) and (II) were tested to assess their inhibitory activity toward PAP. The structure–activity relationships observed with the enzyme inhibition were consistent with those already observed for the inhibitory activity of parent compounds (I) and (II) toward TNAP [38].

Moreover, the propan-2-ylamino-substituted compound (I) and the derivative (II), which bears the dimethylamino group, exhibited excellent stronger competitive (I) and weaker competitive (II) inhibitory activity toward PAP, respectively.

**Figure 2:**
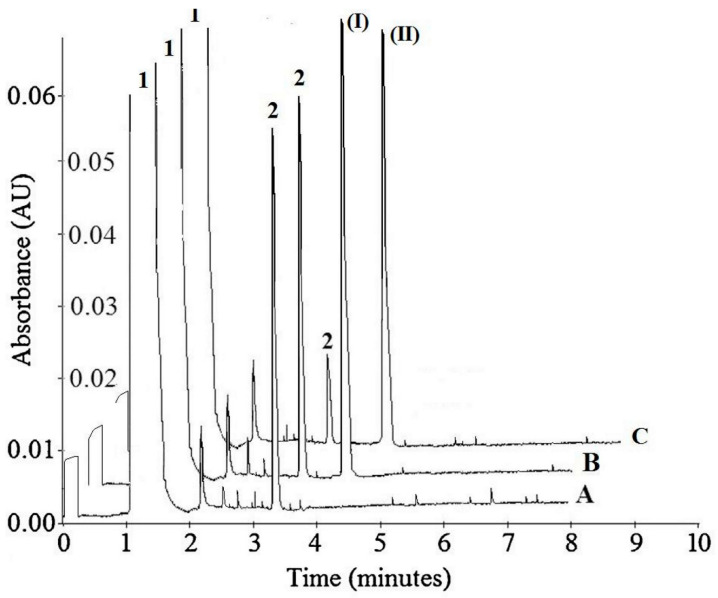
Representative electrophoregrams of: (A) 1.44 mM of p-nitrophenyl phosphate (PN-TE) (1) and 0.018 mM of the reaction product p-nitrophenol (PN-OL) (2) in 2 mL of incubation sample; (B) 1.44 mM of PN-TE (1) and 0.018 mM of the reaction product PN-OL (2) in the presence of 0.05 mM of (**I**) in 2 mL of incubation sample; and (C) 1.44 mM of PN-TE (1) and 0.009 mM of the reaction product PN-OL (2) in the presence of 0.05 mM of (**II**) in 2 mL of incubation sample.

CE enabled us to carry out selective monitoring of p-nitrophenol (PN-OL), p-nitrophenyl phosphate (PN-TE), and compound (I) and compound (II), as well as to eliminate any interference with the endogenous components originating from the serum samples (Figure 2). 

**Figure 3:**
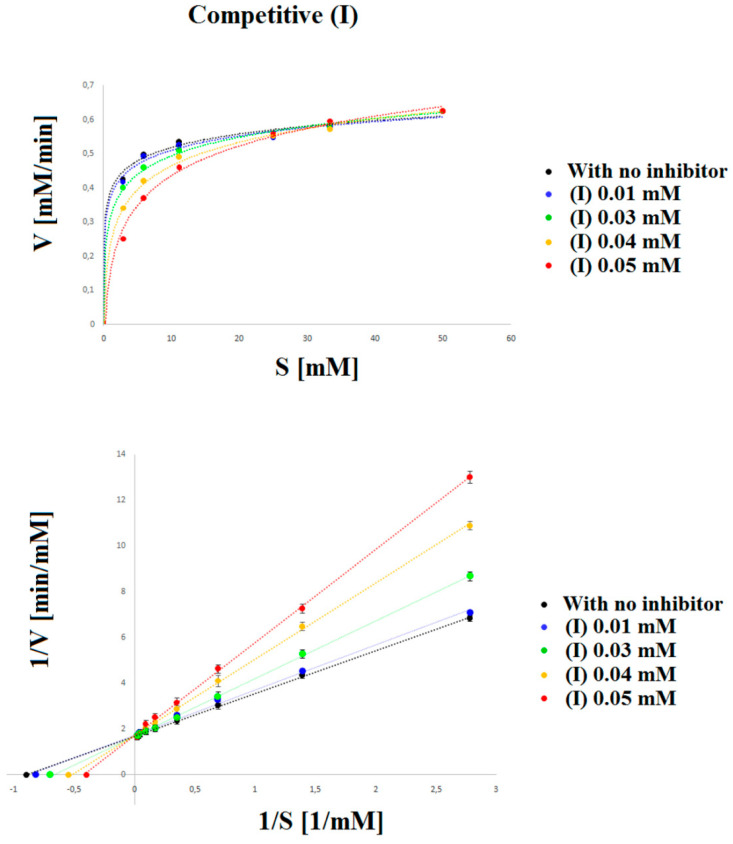
Michaelis–Menten and Lineweaver–Burk plots for the reaction of acid phosphatase with p-nitrophenyl phosphate inhibited by: (•) 0.00 mM, (•) 0.01 mM, (•) 0.03 mM, (•) 0.04 mM and (•) 0.05 mM (I). The figure shows three straight lines with different intersection points with the 1/S axis and a common intersection with the 1/V axis. Such a system indicates the competitive type of inhibition. The larger the slope of the curve, the stronger the competitive inhibitor of the tested compound is.

**Figure 4:**
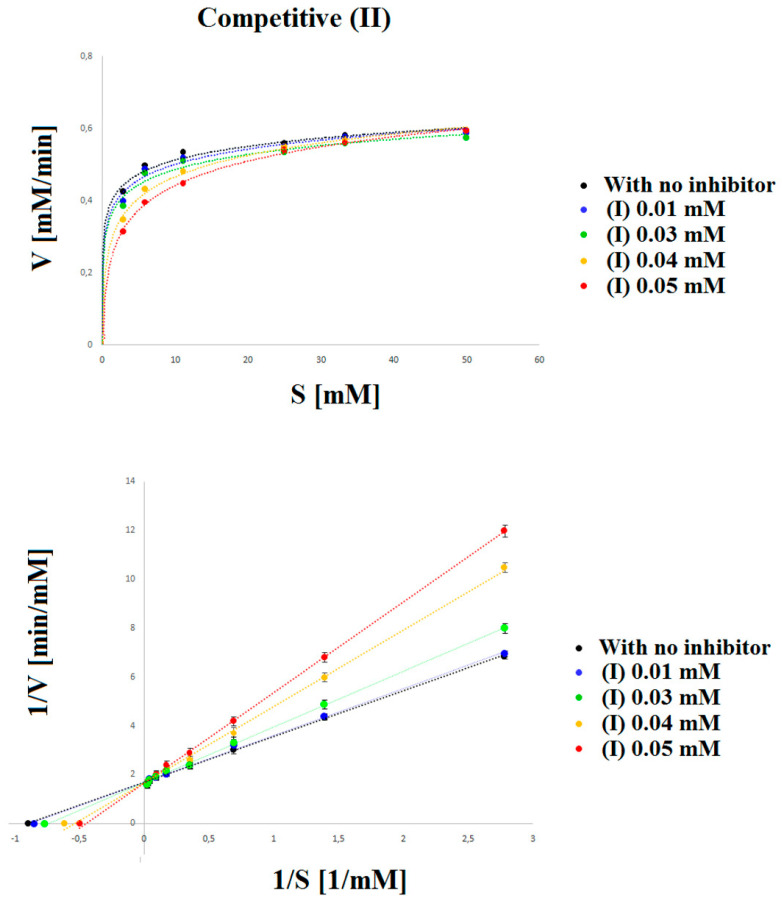
Michaelis–Menten and Lineweaver–Burk plots for the reaction of acid phosphatase with p-nitrophenyl phosphate inhibited by: (•) 0.00 mM, (•) 0.01 mM, (•) 0.03 mM, (•) 0.04 mM and (•) 0.05 mM (II). The figure shows three straight lines with different intersection points with the 1/V axis and a common intersection with the 1/S axis. Such a system indicates the competitive type of inhibition. The larger the slope of the curve, the stronger the competitive inhibitor of the tested compound is.

In this work, we used, to a large extent, the method developed earlier by us [38]; however, to obtain better separation parameters, we changed the buffer and voltage value. For the development of this method, we optimized the buffer pH, the separating BGE concentration, wavelength, temperature, and voltage. The separation of all compounds was investigated at three pH values (2.5, 3.0, and 3.5), three temperatures (20, 25, and 30 °C), four voltages (10, 15, 17, and 20 kV), three concentrations of separating BGE (20, 30, and 50 mM), and four wavelengths (200, 214, 280, and 300 nm). All these separation systems were tested to achieve the best separation parameters, resolution, and analysis time for all components in the reaction mixture. The best separation of PN-OL, PN-TE, and compounds (I) and (II) was obtained with 50 mM tetraborate buffer (pH 2.5). The measurements were taken at 200 nm with a fused-silica capillary (effective length: 20 cm, diameter: 50 μm). The capillary temperature was 20 °C, which allowed the best resolution to be achieved. Separation was carried out at 17 kV. Each sample was added to the capillary under hydrodynamic injection. Average detection time for PN-TE, PN-OL, compound (I), and compound (II) was 1.09, 3.42, 4.12, and 4.45 min, respectively (Figure 2). The constructed curves were linear over the concentration range of 0.36–46.00 mM used for PN-TE. The basic enzymatic activity was investigated using nine concentrations of reaction substrate (PN-TE) (0.36, 0.72, 1.44, 2.88, 5.75, 11.50, 23.00, 34.50, and 46.00 mM) in the presence of the enzyme (PAP). The effect of inhibition of compounds (I) and (II) was determined by an enzymatic kinetics study in a system containing successively increasing concentrations of the substrate at a constant concentration (0.05 mM) of inhibitor (I) and (II). The correlation coefficient for PN-TE in the absence of inhibitors was found to be 0.9997, and the slope of the curve was 1.766. With 0.04 mM compound (I) and 0.05 mM compound (II), the correlation coefficients were determined at 0.998–0.999, and the curve slopes at 3.125 and 4.019 for compound (I) and 2.191 and 2.658 for compound (II), based on the inhibition type and inhibitory strength (Table 1, Figure 3 and Figure 4).

Linearity of the reaction for each substrate -product concentrationhas been shown in Figure S1.

The statistical power for each dataset and each value of the kinetic parameter was achieved by measuring six times each value obtained for the nine concentrations of substrates and reaction products in the system without inhibitors and for four systems in the presence of 0.01 mM, 0.03 mM, 0.04 mM and 0.05 mM inhibitor (I) or (II). After exceeding the value of 0.05 mM, there was no further increase in inhibition, therefore the value of 0.05 mM was considered the cutoff value for both inhibitors.

The inhibitory effects of compounds (I) and (II) on PAP are shown in the form of electrophoregrams in Figure 2, with the detailed data presented in Tables S1 and S2 in the Supplementary Materials section.

Lineweaver–Burk curves intersecting at one common point on the 1/Vmax axis indicate the stronger competitive type of inhibition (compound (I)), whereas curves having one common intersection on the 1/S axis indicate the weaker competitive type of inhibition (compound (II)) (Figure 3 and Figure 4). The Km and Vmax values were determined from the equation y = ax + b in the case of each line. By comparing these values for the system without and with inhibitor, the inhibition type was identified (Table 2).

Inhibition constants were also calculated for the two enzymatic systems. Using equation Ki = IC_50_/([S]/Km + 1), Ki was calculated at 0.786 mM and 0.924 mM for the competitive (I) and competitive (II) inhibitor, respectively.

To determine the type and strength of PAP inhibition by compounds (I) and (II) and the strength of affinity of inhibitors (I) and (II) to the enzyme, we estimated the differences in the slope angles of straight lines, values of Michaelis–Menten constant (Km), and inhibition constants (Ki) between the control systems (without an inhibitor) and the systems containing inhibitors (I) and (II) at 0.05 mM concentration (Table 2).

**Figure 5:**
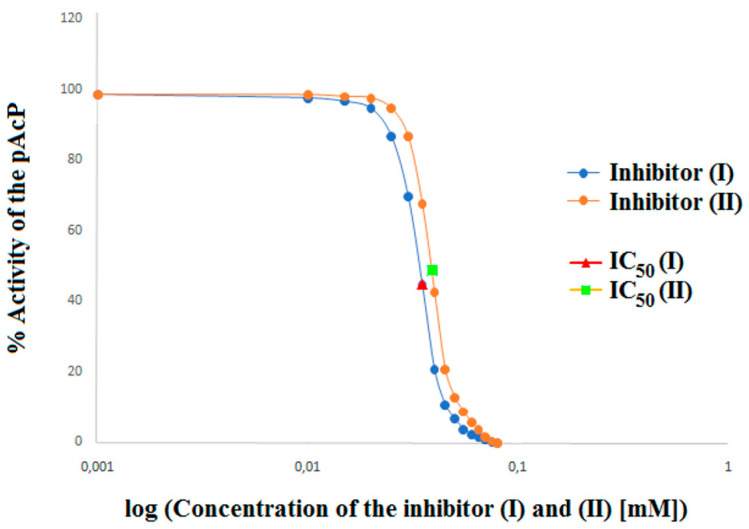
Determination of half-maximal inhibitory concentration (IC_50_) for compound (I) and (II).

In order to accurately determine the effect of inhibitors (I) and (II) on prostatic acid phosphatase, apart from the parameters defining the type of inhibition, the affinity of the inhibitor to the enzyme and the maximum speed, the parameters of the inhibition constant (Ki), turnover number (kcat), catalysis rate (kcat/Km) and half-maximal inhibitory concentration (IC_50_) of PAP in the presence of a competitive (I) and competitive (II) inhibitor were also determined (Table 3). Both the tested aminoalkanol derivatives inhibited PAP, with IC_50_ values of 37 μM for compound (I) and 39 μM for compound (II) (Table 3, Figure 5).

**Figure 6:**
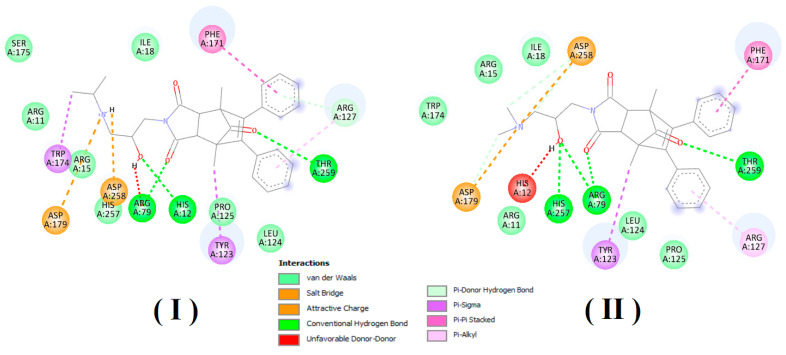
Structure of the active site of prostatic acid phosphatase and potential types of interactions between the competitive inhibitor (**I**) and (**II**) and amino acids present in the active site of the enzyme.

**Figure 7:**
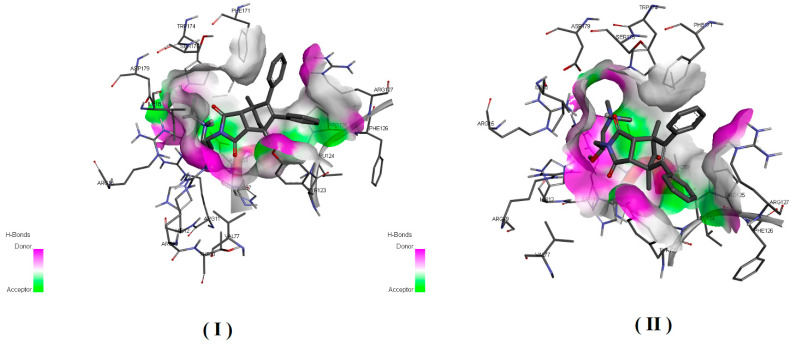
Structure and bioactive conformation of the inhibitor in a binding pocket of prostatic acid phosphatase.

Taking into account the structure of the acid phosphatase and the structures of a competing inhibitor molecules, we also analyze the possibility of their interaction (Figure 6 and Figure 7) with the active site of enzyme.

In order to estimate the potential bioactive conformation of the compounds and possible interactions stabilizing the protein–ligand interaction, molecular docking methods were used. Both compounds (I and II) as well as ligand (alpha-benzyl-aminobenzyl-phosphonic acid) crystalized in active site of PAP in 1nd5 PDB structure (classified as strong inhibitor) were docked in the crystallographic structure of the human prostate acid phosphatase active site using the Autodock Vina program. The obtained values of estimated binding free energy for compound (I) were slightly higher and amounted to −8.0 kcal/mol and for compound (II) −7.8 kcal/mol, what was in agreement with the experimental data. Both compounds scored higher in comparison to the crystallized ligand for which binding interaction was estimated as −7.5 kcal/mol.

Analysis of bioactive conformations showed that both compounds adopt nearly identical conformations at the binding site. In the case of both compounds’ non-polar part this was stabilized in the active pocket by interactions with, TYR123, LEU124, ARG127, PHE171 and polar fragments form hydrogen bonds with ARG79, THR259 HIS257.

Predicted differences was due to structural diversity within the amino group and resulting from the occurring hydrogen interactions with polar amino acids at the binding pocket. In both cases, the protonated amino group formed an intramolecular hydrogen bond with the carbonyl group. The differences were due to the presence of an intermolecular hydrogen bond between ASP258 and the second hydrogen atom bonded to the amine group which was present in compound (I) but not possible for compound (II) due to the methylation. Additionally unfavorable donor–donor interaction with HIS12 occurred for compound II. The receptor–ligand interaction pattern is depicted in Figure 6 and bioactive conformation in a binding pocket is presented in Figure 7.

The simultaneous formation of two bonds between the inhibitor (I) and (II) and the active site proves the high binding strength and affinity of this inhibitor to PAP, as shown by the experimental results presented in Table 3.

## 3. Discussion

Using the electrophoresis method developed, we investigated the necessary kinetic parameters of the PAP-catalyzed enzymatic reaction in the presence and absence of compounds (I) and (II) in a very short time. The method was found to be characterized by very good validation parameters, which enabled its use in studying enzymatic kinetics. From the results obtained, we determined the type of inhibition and the inhibitory strength of compounds (I) and (II) as well as their affinity to the enzyme (PAP).

The results indicated compound (I) to be a stronger competitive inhibitor and compound (II) as a weaker competitive inhibitor of PAP. All data and the calculations of the slope angles of straight lines in the course of enzymatic reactions and the Km, Ki, kcat, kcat/Km, IC_50_ and scoring function parameters (Table 3) clearly showed that compound (I) was a stronger competitive inhibitor of PAP than compound (II). The results also showed that the affinity of compound (I) to the enzyme was greater than that of compound (II), as indicated by the values of Ki and Km (Table 3).

The comparison of the slope angles of straight lines for compound (I) and (II) showed that compound (I) was a 1.02 times more potent competitive inhibitor of PAP than compound (II). The slope of the line for inhibitor (I) was 1.312 degrees greater than for inhibitor (II), which also indicated a greater degree of inhibition of compound (I) compared to compound (II).

Comparing of the Km values for the system without inhibitor (1.124 mM) with systems containing inhibitor (I) (2.429 mM) and inhibitor (II) (2.216 mM) and Vmax values (0.600 mM/min, 0.597 mM/min and 0.598 mM/min, respectively), it is clearly seen that in both cases the Vmax values remain practically unchanged, while the Km values clearly increase relative to the reference system (without the inhibitor). This clearly indicates the competitive type of inhibition of compounds (I) and (II), and comparison of the Km value for inhibitor (I) with the Km value for inhibitor (II) shows that compound (I) is a stronger competitive inhibitor of PAP than compound (II).

Differences in the inhibitory potency of PAP can also be seen when comparing the values of inhibition constants (Ki) characterizing the strength of the inhibitor’s affinity for the enzyme. Low Ki values in all cases indicate a high affinity of inhibitors (I) and (II) to PAP.

Comparing the values of the Ki constants for the competitive (I) (0.786 mM) and competitive (II) (0.924 mM) inhibitor, it can be seen that affinity of the inhibitor (I) to the enzyme was 1.176 times stronger than competitive inhibitor (II). The difference between the Ki value for inhibitor (I) and (II) was 0.138 mM which also indicated a stronger affinity of compound (I) to the PAP compared to compound (II).

The values of the catalytic constants (kcat), characterizing the turn over number of the enzyme, were 1.299 × 10^−2^ s^−1^ for the enzyme inhibited by a competitive inhibitor (I) and 1.304 × 10^−2^ s^−1^ for non-inhibited enzyme. It follows from the above data that the turnover number of PAP and thus the number of substrate molecules converted into the reaction product in the presence of a competitive inhibitor (I) was 1.004 times less than in the presence of the non-inhibited enzyme, and the difference between kcat values of the inhibited and uninhibited enzyme was 0.005 × 10^−2^ s^−1^. In the case of a competitive inhibitor (II) the values of the catalytic constants (kcat) were 1.300 × 10^−2^ s^−1^ for the enzyme inhibited by competitive inhibitor (II) and 1.304 × 10^−2^ s^−1^ for the non-inhibited enzyme; therefore, turnover number in the presence of competitive inhibitor (II) was 1.003 times smaller than in the presence of the non-inhibited enzyme, and the difference between kcat for inhibited and non-inhibited enzyme was 0.004 × 10^−2^ s^−1^.

Thus, when comparing the kcat values for inhibitor (I) and (II) and the differences between them, it is clearly seen that compounds (I) and (II) have no major influence on the turnover number of the enzyme and confirm the inhibition competency of both compounds.

Comparison of the specificity constants (kcat/Km) for the enzyme in the presence of compound (I) (0.549 × 10^−2^ M^−1^ s^−1^) and the non-inhibited enzyme (1.160 × 10^−2^ M^−1^ s^−1^) showed that in the presence of a competitive inhibitor (I) the catalytic efficiency of the enzyme was 2.11 times lower than the non-inhibited enzyme. Comparison of the specificity constants (kcat/Km) for the enzyme in the presence of compound (II) (0.595 × 10^−2^ M^−1^ s^−1^) and the non-inhibited enzyme (1.160 × 10^−2^ M^−1^ s^−1^) showed that in the presence of a competitive inhibitor (II) the catalytic efficiency of the enzyme was 1.95 times lower than the not inhibited enzyme.

The differences in kcat/Km values between the inhibitor (I)-non-inhibited enzyme system and the inhibitor (II)-non-inhibited enzyme system were 0.611x10^−2^ M^−1^ s^−1^ and 0.565 × 10^−2^ M^−1^ s^−1^, respectively. It follows that the catalytic capacity of the enzyme (PAP) and thus the enzyme’s overall ability to convert substrate to product is lower for inhibitor (I) than for inhibitor (II), which is further evidence that compound (I) is a stronger competitive inhibitor of PAP than compound (II).

The influence of the type of substituent is also clearly visible when comparing the IC_50_ values for inhibitors (I) (37 µM) and (II) (39 µM). The comparison of these values shows that compound (I) is 1.05 times more potent PAP inhibitor than compound (II). Such a small difference is due to the small, but well registered by our method, difference between the substituents present in the main structure of compounds (I) and (II).

The obtained values of the binding free energy for compound (I) were slightly higher and amounted to −8.0 kcal/mol and for compound (II) −7.8 kcal/mol, which also confirmed the stronger bond of compound (I) to PAP than compound (II).

There are many papers describing the influence of various compounds on prostatic acid phosphatase. The most popular are works describing the effects of inhibitors such as benzylphosphonic acids [42,43] And L (+)-Tartrate [44].

Benzylphosphonic acid and its derivatives exhibit a very broad spectrum of prostatic acid phosphatase inhibitory potency in the IC_50_ range from >500,000 nM do 4 nM [42,43].

In the case of aminoalkanol derivatives, the IC_50_ values are 37,000 nM for compound (I) and 39,000 nM for compound (II), respectively.

Comparing the IC_50_ values for benzylphosphonic acid and compounds (I) and (II) it can be shown that compound (I) is a 13.5 times more potent PAP inhibitor and compound (II) is a 12.8 times more potent PAP inhibitor than benzylphosphonic acid.

On the other hand, taking into account the IC_50_ value (4 nM) obtained for α-benzylamino substituted analogue of benzylphosphonic acid, compounds (I) and (II) show 9250 and 9750 times less inhibitory potency, respectively.

Comparing the inhibitory potency of all benzylphosphonic acid derivatives with the inhibitory potency of compounds (I) and (II), it can be stated that they are ranked 48th and 50th in terms of potency among 65 benzylphosphonic acid derivatives [42,43]. The inhibition potency of compound (I) (IC_50_ = 37,000 nM) is identical with the inhibition potency of R_1_-(4-methoxy) -R_2_ (phenyl) analogue of benzylphosphonic acid. In contrast, the inhibitory potency of compound (II) (IC_50_ = 39,000 nM) occupies a place between R_1_-(2-methyl) -R_2_ (phenyl) analogue (IC_50_ = 38,000 nM) and R_1_-(4-trifluoromethyl) -R_2_ (phenyl) analogue (IC_50_ = 41,000 nM) of benzylphosphonic acid.

Another type of PAP inhibitor is the widely described L (+)-Tartrate [44], whose inhibitory potency (IC_50_) for prostatic acid phosphatase is 53,000 nM and the affinity strength (Ki) is 29,000 nM.

Based on the IC_50_ values of 37,000 nM for inhibitor (I) and 53,000 nM for L (+)–tartrate [44], it can be seen that inhibitor (I) is a 1.43 times more potent PAP inhibitor while inhibitor (II) with IC_50_ value of 39,000 nM is 1.36 times more potent as an inhibitor of PAP than L (+)-tartrate.

While comparing the values of the affinity strength (Ki = 786,000 nM) for inhibitor (I) and (Ki = 924,000 nM) for inhibitor (II) with the constant Ki = 29,000 nM for L (+)–tartrate inhibitor [44], it can be seen that in this case the affinity of inhibitors (I) and (II) to PAP is smaller and amounts to 27.1 and 31.8 times less, respectively. This interesting comparison may indicate a greater intrinsic activity of inhibitors (I) and (II) than L (+)-tartrate in relation to PAP.

Summarizing the results of our research, it can be stated that the CE method developed by us allows for the capture and study of subtle differences between the structure of compounds (I) and (II) and the type, inhibition potency, specificity and affinity of both compounds to PAP.

The results also clearly show that the compounds (I) and (II) are stronger PAP inhibitors than L (+)-tartrate and show very similar inhibitory potency compared to some derivatives of benzylphosphonic acid and the type and strength of inhibition as well as the affinity to the prostatic acid phosphatase depends on the type of the substituent present in the main structure of compounds (I) and (II).

## 4. Materials and Methods

Part of the methodology is very similar to that described in the previous article [38] because some experimental conditions had to be identical and consistent with the previous ones. Preliminary identification and determination of prostatic acid phosphatase were performed according to the immunoelectrophoresis procedure described below [43].

Glass slides, 50 by 75 mm, coated with dilute agar solution were dried in the oven. We poured 10 mL of 1% agarose in 0.025 M borate buffer, pH 8.2, on the coated slides and stored them in moist chambers in the cold. Parallel rows of cells, 8 mm in diameter, containing 50 μL of solution, were divided. Prostatic acid phosphatase, patients’ sera was placed in the left well (cathode) and anti-PAP (anti sera in the right well (anode). The electrophoresis was run at 11 ma. per slide for 135 minutes, and the voltage averaged 100 V. The buffer maintained its temperature at 23 °C at the beginning until the end of the run, without any cooling. After washing, the slides were stained by the NFR method at 37 °C. for two hours and then left overnight at room temperature. The next day, the slides were washed in dilute acetic acid and the reaction evaluated. For the determination and measuring of prostatic acid phosphatase, the chemical method was used. Briefly, this method uses sodium thymolphthalein monophosphate as a substrate for measuring acid phosphatase activity in sera and bone marrow. Thymolphthalein lrin is liberated in the reaction and is conveniently measured by increasing the pH of the medium which produces a color that is used for the quantitative and qualitative evaluation of the prostatic acid phosphatase [45].

### 4.1. Principles

**Figure 8:**
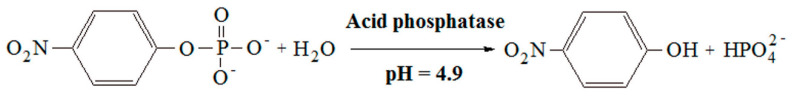
Scheme of the reaction catalyzed by acid phosphatase. The reaction rate was determined by the rate of loss of the amount of substrate (p-nitrophenyl phosphate) and the increase in the amount of product (p-nitrophenol) per unit of time.

The activity of acid phosphatase was measured from the reaction shown in Figure 8. In serum, this enzyme catalyzes the reaction of hydrolysis of a colorless substrate, p-nitrophenyl phosphate (PN-TE), resulting in a yellow-colored product, p-nitrophenol (PN-OL) (Figure 8).

The color intensity of the obtained product was assessed at 200 nm. The results obtained from determining the inhibition type using the following equation are shown on the Lineweaver–Burk coordinate system in Figure 3 and Figure 4:


$$\frac{1}{V}\;=\;\frac{K_{m}}{V_{\max}\left[S\right]}\;+\;\frac{1}{V_{\max}}$$

### 4.2. Reagents and Chemicals

The compounds (I) and (II) were synthesized and chemically analyzed (mass spectrometry and nuclear magnetic resonance spectroscopy) as described previously [37]. The reference compounds had a purity of above 98%, while the remaining analytes used in the performed separations had 99% purity. We used phosphate buffer (obtained from Beckman-Coulter, Brea, CA, USA), PN-TE, PN-OL, deionized water (from Sigma-Aldrich, Darmstadt, Germany), trisodium citrate (from Sigma-Aldrich, Steinheim, Germany), NaOH, and Bovprec Control 2 (serum standard, bovine serum-based; obtained from Randox, Warsaw, Poland).

### 4.3. Instrumentation

A Beckman Coulter P/ACE MDQ CE system was used for electrophoretic analysis. The instrument had an autosampler along with a UV–Visible detector. All CE parameters were controlled using Karat software (v. 32). Separation was carried out using eCAP fused-silica capillary (total length: 30 cm, effective length: 20 cm, inner diameter: 50 μm, outer diameter: 375 μm).

### 4.4. Capillary Electrophoresis (CE) Conditions

Electrophoretic separations of PN-TE, PN-OL, and compounds (I) and (II) by CE was performed using 50 mM tetraborate buffer as a background electrolyte (BGE), at pH 2.5 adjusted with 100 mM hydrochloric acid. Experimental conditions were set as described in our previous work [38]. The samples were injected under 5 psi pressure for 3 s. The experiments were performed under constant current conditions (85 μA). The separation temperature was 20 °C, and the detection wavelength was set at 200 nm. Before the assay, the capillary was conditioned with 0.1 M NaOH for 10 min at 10 psi and with H_2_O for 10 min at 10 psi. Then, it was electroconditioned with the running buffer for 20 min at +10 kV and pressure-conditioned with the running buffer for 10 min at 10 psi. Before each injection, the capillary was washed with 1 M NaOH for 2 min, then with H_2_O for 2 min, and finally with BGE for 2 min. At the end of each working day, the capillary was rinsed for 10 min with 0.1 M NaOH, 10 min with BGE, and 10 min with deionized water at 15 psi. Then, the capillary was exposed to air for 10 min at 20 psi and left empty.

Sample mixtures containing citrate buffer (pH 4.9), compound (I) or (II) solution, serum, and NaOH solution were introduced automatically by pressure injection (with 2 psi pressure for 5 s on the sample solution). At the day’s end, vials with anode and cathode buffer were emptied and running buffer was filled again at the start of the next day before the analysis. The detector wavelength was fixed at 200 nm. The appropriate parameters for analyzing PN-OL were as follows: tetraborate buffer (pH 2.5) of 50 mM concentration, 20 °C temperature, and 17 kV voltage.

### 4.5. Preparation of Stock and Working Standards

Primary stock standard solutions were prepared for compound (I) and compound (II) in deionized water (concentration of each = 50 mM). Primary stock standard solutions were also prepared for PN-TE separately using deionized water, with 160 mg of PN-TE dissolved in 40 mL of citrate buffer (50 mM, pH 4.9), achieving 46.00 mM concentration. The prepared PN-TE solution was diluted with citrate buffer (50 mM), and mixed working standard solutions were obtained (concentrations: 34.50, 23.00, 11.50, 5.75, 2.88, 1.44, 0.72, and 0.36 mM) as described previously [38].

### 4.6. Sample Preparation

In nine test tubes, 1.7 mL citrate buffer (50 mM concentration, pH 4.9) containing PN-TE (concentrations of 0.36–46.00 mM), 0.02 mL substance (I) or (II) solution, and 0.08 mL H_2_O was added. To another tube (zero sample, tube 10), 1.8 mL of citrate buffer (50 mM concentration, pH 4.9) containing PN-TE (0.36 mM concentration) and 0.08 mL H_2_O was added. The tubes containing solutions were preincubated for 5 min at 37 °C. Then, 0.1 mL serum was introduced into tubes 1–9, at an interval of 1 min. All the 10 samples were incubated for 15 min at 37 °C. After incubation, 0.1 mL of NaOH (2 M) was introduced to these analytical samples at an interval of 1 min. Finally, the analytical samples were added to the capillary.

To assess possible interaction, molecular docking experiments were performed using the PyRx docking tool [46] via the Autodock VINA software [47,48]. The three-dimensional (3D) crystal structure of human prostate acid phosphatase (PAP) (PDB code: 1nd5) was obtained from the RCSB Protein Data Bank. The crystallographic structure was resolved with a resolution of 2.9A in a complex with the strong inhibitor of the phosphate derivative, alpha-benzyl-aminobenzyl-phosphonic acid.

The three-dimensional (3D) structure (PDB format) of PAP has been analyzed and prepared for docking studies with the PyMOL software (The PyMOL Molecular Graphics System, Version 2.0 Schrödinger, LLC). The active site was identified by the presence of the inhibitor. The PDB PAP file was uploaded to the PyRx utility linked to the Autodock VINA. The macromolecule was converted to the pdbqt format, which automatically removes solvent particles, adds hydrogen and performs Gasteiger charges calculations. The center of the mesh was placed in active sites based on the position of selected amino acids classified as a binding site pocket (ARG 79, TYR 123, ILE18, HIS257). No constraints were used in the docking proces. The default exhaustive value was used to maximize the conformational binding analysis. The generated docked complexes were selected based on binding affinity values (kcal/mol) and binding interaction patterns (hydrogen, hydrophobicity, and electrostatic) were analyzed for higher scored conformation. Graphical representations of all docked complexes were made using the Discovery studio visualizer version 4.0 (BIOVIA, San Diego, CA, USA).

## 5. Conclusions

Acid phosphatase is an enzyme involved in a wide spectrum of biochemical processes. However, the enzyme plays the most important role in prostate diseases such as prostate hyperplasia or prostate cancer and its regulation can have a significant impact on the development and treatment possibilities of these diseases [4,21].

In addition, acid phosphatase participates in bone formation, mineralization, and breakdown, and thus is an essential factor in the regulation of bone diseases such as osteoporosis and osteomalacia [6,22,23].

As several medicinal substances can interact with different enzyme systems, this study investigated the influence of newly patented aminoalkanol derivatives (I) and (II) of 1,7-dimethyl-8,9-diphenyl-4-azatricyclo[5.2.1.02,6] dec-8-ene-3,5,10-trione, which have potential anticancer activity, on the activity of prostatic acid phosphatase.

We used the CE method developed earlier by us [38]. This method allowed us to analyze the type of inhibition through the determination of the concentrations of substrate (PN-TE) and reaction product (PN-OL) of acid phosphatase, in the presence of compound (I) or (II) in the serum samples.

As aminoalkanol derivatives were not recognized as PAP inhibitors and not reported in the literature so far, we attempted to analyze their effect on the activity of PAP. Our study showed that PAP inhibition can be assessed simply with an in vitro drug metabolic system using a CE analysis. The results showed that the studied derivatives exerted different effects on PAP. One of the analyzed compounds (I) was identified as a stronger competitive inhibitor, and the other (II) as a weaker competitive inhibitor of the PAP. Furthermore, the findings indicated that the type of substituent present in the main chemical molecule determines the inhibition type, and that the competitive inhibition of compound (I) is stronger than the competitive inhibition of compound (II). The investigations performed also showed differences in the inhibitory potency and affinity of compounds (I) and (II) to prostatic acid phosphatase (PAP).

The described properties of compounds (I) and (II) and the knowledge of their effect on the activity of prostatic acid phosphatase can be of use in designing therapy for many diseases. In addition, such an analysis of properties can indicate the different possibilities of influencing the regulation of specific biochemical processes, which can be considered important information on substances to be used in medicine.

## Figures and Tables

**Table 1:** Regression equation and quantification for p-nitrophenyl phosphate for 6 replicates for each sample (*n* = 6) in the concentration range 0.36–46.00 mM, in the presence of inhibitors (I) and (II) at a concentration of 0.01, 0.03, 0.04 and 0.05 mM.

Concentration Inhibitors (I) and (II) (mM)	Linearity Range of Substrate (P-Nitrophenyl Phosphate) [mM]	R^2^	RSD (%)	LOD (mM)	LOQ (mM)	Regression Equation	Standard Deviation	
							Slope	Intercept
0	0.36–46.00	0.9997	2.68	0.20	0.36	y = 1.870x + 1.664	±0.041	±0.029
(I) 0.01	0.36–46.00	0.9996	2.85	0.22	0.36	y = 1.976x + 1.739	±0.049	±0.033
(I) 0.03	0.36–46.00	0.9995	2.91	0.22	0.36	y = 2.530x + 1.767	±0.061	±0.036
(I) 0.04	0.36–46.00	0.9996	2.93	0.22	0.36	y = 3.321x + 1.740	±0.062	±0.039
(I) 0.05	0.36–46.00	0.9996	2.98	0.25	0.36	y = 4.069x + 1.675	±0.056	±0.038
(II) 0.01	0.36–46.00	0.9995	3.21	0.23	0.36	y = 1.919x + 1.698	±0.059	±0.041
(II) 0.03	0.36–46.00	0.9994	2.19	0.22	0.36	y = 2.274x + 1.689	±0.051	±0.040
(II) 0.04	0.36–46.00	0.9996	3.35	0.23	0.36	y = 3.131x + 1.669	±0.064	±0.042
(II) 0.05	0.36–46.00	0.9996	3.42	0.25	0.36	y = 3.701x + 1.670	±0.057	±0.040

LOD—Limit of detection; LOQ—Limit of quantification; RSD—Relative standard deviation.

**Table 2:** Determination of the inhibition type for PAP by comparing the Km and Vmax values between the control systems without inhibitor and the systems containing inhibitors (I) and (II) at a concentration of 0.05 mM. An increase of the Km value with a constant Vmax value in comparison to the basic system indicates the stronger competitive type of inhibition (compound (I)). A constant Km value with a reduced Vmax value in comparison to the basic system indicates the weaker competitive type of inhibition (compound (II)). The results show the mean values for the concentration of 0.05 mM inhibitors (I) and (II) for the 9 substrate concentrations. Each measurement was performed six times (*n* = 6).

	Control System (without Inhibitor)	System with Inhibitor (I)			System with Inhibitor (II)		
		Mean	SD	%RSD	Mean	SD	%RSD
Km [mM]	**1.124**	**2.429**	0.041	1.742	**2.216**	0.020	1.944
Vmax [mM/min]	**0.600**	**0.597**	0.007	1.242	**0.598**	0.079	1,261
Type of inhibition		Competitive (I)			Competitive (II)		

**Table 3:** Parameters characterizing the inhibitory strength of PAP and TNAP by compounds (I) and (II) (value of straight angle), the affinity strength of compounds (I) and (II) to both enzymes (Km and Ki values), catalytic constants (kcat), catalytic efficiencies (kcat/Km) and half-maximal inhibitory concentration (IC_50_).

	ENZYME					
	PAP					
	With No Inhibitor	SD	Competitive Inhibitor (I)	SD	Competitive Inhibitor (II)	SD
Value of straight angle	61.860	0.185	76.191	0.463	74.879	0.304
Km [mM]	1.124	0.031	2.429	0.076	2.216	0.035
Ki [mM]			0.786	0.002	0.924	0.002
kcat [s^−1^]	1.304 × 10^−2^	5.211 × 10^−4^	1.299 × 10^−2^	5.790 × 10^−4^	1.300 × 10^−2^	5.579 × 10^−4^
kcat/Km [M^−1^s^−1^]	1.160 × 10^−2^	2.643 × 10^−4^	0.549 × 10^−2^	2.290 × 10^−4^	0.595 × 10^−2^	4.710 × 10^−4^
IC_50_ [mM]			0.037	0.001	0.039	0.001
Scoring function [kcal/M]			8.0		7.8	

## Data Availability

Data Availability Statements in section “MDPI Research Data Policies” at https://www.mdpi.com/ethics (accessed on 1 June 2021).

## Supplementary Materials

The following are available online at https://www.mdpi.com/article/10.3390/ijms222111761/s1.
