# Supplementary material for: Kinetic Studies of Newly Patented Aminoalkanol Derivatives with Potential Anticancer Activity as Competitive Inhibitors of Prostate Acid Phosphatase
Source: Int J Mol Sci. 2021 Oct 29;22(21):11761. doi: 10.3390/ijms222111761 (PMC8584256; doi:10.3390/ijms222111761)
Supplement: Supplementary file 1 [file ijms-22-11761-s001.zip › ijms-1392157-SI.pdf]

International Journal of *Molecular Sciences*

MDPI

Supplementary Materials

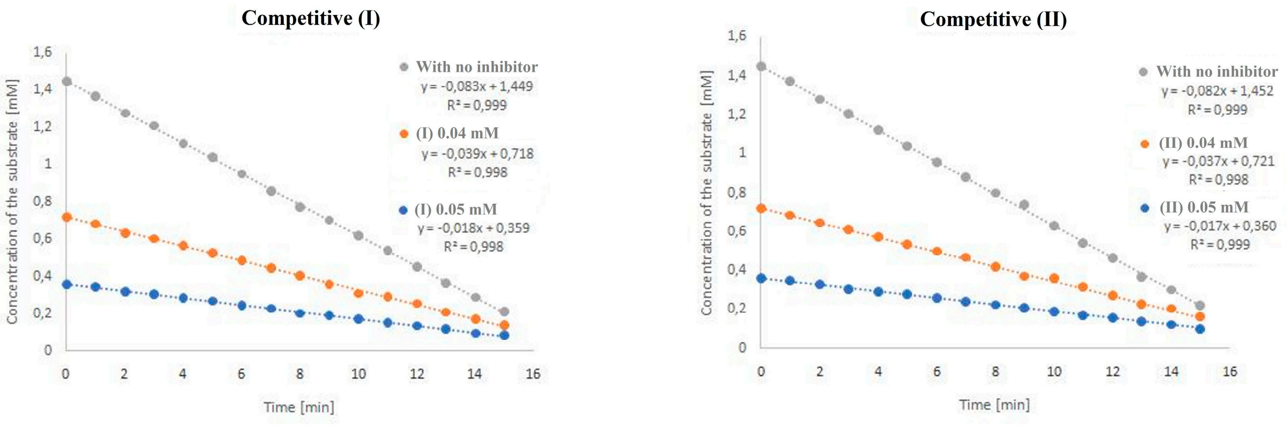

Figure S1: Linearity of the reaction for each substrate -product concentration.

Table S1: Competitive inhibition. Values characterizing the course of the reaction in the presence of the inhibitor (I) of acid phosphatase. The data present the changes of peak area corresponding to the increase in the amount of reaction product (p-nitrophenol) and reaction speed (V_0 and $1/V_0$) in the system without inhibitor (I) ($C_{0.00}$) and with inhibitor (I) at concentrations of 0.04 mM ($C_{0.04}$) and 0.05 mM ($C_{0.05}$) and the other concentration from 0.00 mM to 0.05 mM. The changes in enzymatic reaction parameters were registered in the presence of the substrate (p-nitrophenyl phosphate) at specific concentrations in the range of 0.36–46.00 mM.

Substrate concentration (p-nitrophenyl phosphate) [S] (mM)	$\frac{1}{[S]}$	Peak area [PA]								
		**$C_{0.00}$**			**$C_{0.04}$**			**$C_{0.05}$**		
		Mean	SD	%RSD	Mean	SD	%RSD	Mean	SD	%RSD
0.36	2.78	47397	995.3	2.1	37296	1118.8	3.0	34465	999.5	2.9
0.72	1.39	86918	1912.2	2.2	69900	2027.1	2.9	60570	1635.4	2.7
1.44	0.69	122677	2330.9	1.9	124991	3124.8	2.5	98051	2451.3	2.5
2.88	0.35	163009	2934.2	1.8	136426	2864.9	2.1	136402	3273.7	2.4
5.75	0.17	197285	3945.7	2.0	171670	3261.7	1.9	171624	3604.1	2.1
11.50	0.09	220255	3964.6	1.8	213647	3845.6	1.8	211443	4228.9	2.0
23.00	0.04	235993	4483.9	1.9	231623	4169.2	1.8	229438	4129.9	1.8
34.50	0.03	238548	4055.3	1.7	236359	3781.7	1.6	236356	4254.4	1.8
46.00	0.02	239660	4074.2	1.7	239660	4313.9	1.8	239660	4074.2	1.7
		Speed reaction = amount of the produced product/min								
		Amount of the product (p-nitrophenol) [mM/min]								
		**$C_{0.00}$**			**$C_{0.04}$**			**$C_{0.05}$**		
		Mean	SD	%RSD	Mean	SD	%RSD	Mean	SD	%RSD
		0.050	0.001	2.5	0.038	0.001	2.9	0.036	0.001	2.8
		0.089	0.002	2.5	0.073	0.002	2.8	0.064	0.002	2.8
		0.128	0.003	2.3	0.130	0.003	2.6	0.103	0.003	2.7
		0.171	0.004	2.4	0.141	0.003	2.4	0.139	0.004	2.6
		0.206	0.005	2.2	0.180	0.004	2.3	0.178	0.004	2.2
		0.229	0.005	2.1	0.222	0.005	2.3	0.219	0.005	2.4
		0.247	0.004	1.8	0.242	0.005	2.1	0.240	0.005	2.2
		0.249	0.004	1.6	0.247	0.004	1.7	0.247	0.004	1.8

	0.251	0.004	1.7	0.251	0.005	1.8	0.251	0.005	1.8
				V_0 [mM/min]					
	**$C_{0.00}$**			**$C_{0.04}$**			**$C_{0.05}$**		
	Mean	SD	%RSD	Mean	SD	%RSD	Mean	SD	%RSD
	0.14	0.002	1.22	0.09	0.001	0.94	0.07	0.001	1.12
	0.22	0.003	1.54	0.15	0.002	1.25	0.13	0.002	1.18
	0.32	0.006	1.77	0.24	0.004	1.85	0.22	0.004	1.65
	0.42	0.010	2.47	0.35	0.010	2.76	0.32	0.008	2.40
	0.49	0.014	2.76	0.43	0.013	2.95	0.40	0.011	2.72
	0.53	0.018	3.37	0.48	0.017	3.57	0.45	0.016	3.45
	0.55	0.019	3.44	0.55	0.020	3.75	0.54	0.020	3.78
	0.58	0.022	3.87	0.57	0.016	2.89	0.56	0.022	3.85
	0.60	0.024	3.92	0.60	0.024	3.93	0.60	0.024	3.92
				$\frac{1}{Vo}$ [min/mM]					
	**$C_{0.00}$**			**$C_{0.04}$**			**$C_{0.05}$**		
	Mean	SD	%RSD	Mean	SD	%RSD	Mean	SD	%RSD
	6.91	0.08	1.20	10.91	0.10	0.93	13.52	0.15	1.13
	4.35	0.06	1.52	6.49	0.08	1.26	7.59	0.09	1.19
	3.04	0.05	1.78	4.12	0.08	1.86	4.65	0.08	1.64
	2.35	0.06	2.46	2.88	0.08	2.75	3.17	0.08	2.38
	2.01	0.06	2.77	2.31	0.07	2.94	2.52	0.07	2.73
	1.87	0.06	3.38	2.08	0.07	3.58	2.23	0.08	3.46
	1.79	0.06	3.42	1.81	0.07	3.75	1.85	0.07	3.77
	1.72	0.07	3.89	1.75	0.07	2.87	1.78	0.07	3.82
	1.68	0.07	3.92	1.68	0.07	3.94	1.68	0.07	3.91

		Peak area for inhibitor (I) conentrations **$C_{0.00}$ - $C_{0.05}$** [PA]	
	Mean	SD	%RSD
	34465 - 239660	995.3 - 4074.2	1.6 – 3.0
		Amount of the product (p-nitrophenol) in the presence of inhibitor (I) concentrations **$C_{0.00}$ - $C_{0.05}$** [mM/min]	
	Mean	SD	%RSD
	0.036 - 0249	0.001 – 0.005	1.6 – 2.9
		V_0 in the presence of inhibitor (I) concentrations **$C_{0.00}$ - $C_{0.05}$** [mM/min]	
	Mean	SD	%RSD
	0.07 – 0.60	0.001 – 0.024	0.94 – 3.93
		$\frac{1}{Vo}$ in the presence of inhibitor (I) concentrations **$C_{0.00}$ - $C_{0.05}$** [min/mM]	
	Mean	SD	%RSD
	1.68 – 13.52	0.05 – 0.15	0.93 – 3.94

Table S2. Competitive inhibition. Values characterizing the course of the reaction in the presence of the inhibitor (II) of acid phosphatase. The data present the changes of peak area corresponding to the increase in the amount of reaction product (p-nitrophenol) and reaction speed (V_0 and $1/V_0$) in the system without inhibitor (II) ($C_{0.00}$) and with inhibitor (II) at concentrations of 0.04 mM ($C_{0.04}$) and 0.05 mM ($C_{0.05}$) and the other concentration from 0.00 mM to 0.05 mM. The changes in enzymatic reaction parameters were registered in the presence of the substrate (p-nitrophenyl phosphate) at specific concentrations in the range of 0.36–46.00 mM.

Substrate concentration (p-nitrophenylphosphate) [S] (mM)	$\frac{1}{[S]}$									
		Peak area [PA]								
		**$C_{0.00}$**			**$C_{0.04}$**			**$C_{0.05}$**		
		Mean	SD	%RSD	Mean	SD	%RSD	Mean	SD	%RSD
0.36	2.78	47397	995.3	2.1	34125	887.3	2.6	27490	797.2	2.9
0.72	1.39	86918	1912.2	2.2	59573	1489.3	2.5	50783	1371.1	2.7
1.44	0.69	122677	2330.9	1.9	83382	2084.6	2.5	71881	1509.5	2.1
2.88	0.35	163009	2934.2	1.8	125831	2768.3	2.2	100093	2101.9	2.1
5.75	0.17	197285	3945.7	2.0	151315	3177.6	2.1	117796	2709.3	2.3
11.50	0.09	220255	3964.6	1.8	164469	3289.4	2.0	126959	2666.1	2.1
23.00	0.04	235993	4483.9	1.9	168157	3531.3	2.1	130894	2879.7	2.2
34.50	0.03	238548	4055.3	1.7	182024	3458.5	1.9	139871	2657.5	1.9
46.00	0.02	239660	4074.2	1.7	185235	3704.7	2.0	154681	2629.6	1.7
		Speed reaction = amount of the produced product/min								
		Amount of the product (p-nitrophenol) [mM/min]								
		**$C_{0.00}$**			**$C_{0.04}$**			**$C_{0.05}$**		
		Mean	SD	%RSD	Mean	SD	%RSD	Mean	SD	%RSD
		0.050	0.001	2.5	0.036	0.001	2.8	0.029	0.001	3.1
		0.089	0.002	2.5	0.061	0.002	2.5	0.052	0.001	2.6
		0.128	0.003	2.3	0.087	0.002	2.2	0.075	0.002	2.3
		0.171	0.004	2.4	0.132	0.003	1.9	0.105	0.002	2.1
		0.206	0.005	2.2	0.158	0.003	1.9	0.123	0.003	2.2
		0.229	0.005	2.1	0.171	0.004	2.1	0.132	0.003	2.1
		0.247	0.004	1.8	0.176	0.004	2.2	0.137	0.003	1.9
		0.249	0.004	1.6	0.190	0.004	2.0	0.146	0.003	1.9
		0.251	0.004	1.7	0.194	0.004	2.0	0.122	0.002	2.0
		V_0 [mM/min]								
		**$C_{0.00}$**			**$C_{0.04}$**			**$C_{0.05}$**		

	Mean	SD	%RSD	Mean	SD	%RSD	Mean	SD	%RSD
	0.14	0.002	1.22	0.11	0.002	1.44	0.09	0.001	1.39
	0.22	0.003	1.54	0.18	0.003	1.67	0.16	0.003	1.70
	0.32	0.006	1.77	0.27	0.005	1.93	0.21	0.005	2.26
	0.42	0.010	2.47	0.34	0.008	2.45	0.28	0.007	2.64
	0.49	0.014	2.76	0.38	0.009	2.50	0.32	0.009	2.93
	0.53	0.018	3.37	0.40	0.010	2.63	0.35	0.010	2.98
	0.55	0.019	3.44	0.43	0.011	2.57	0.36	0.011	3.05
	0.58	0.022	3.87	0.48	0.014	3.02	0.38	0.009	2.26
	0.60	0.024	3.92	0.51	0.017	3.36	0.42	0.016	3.75

$\frac{1}{Vo}$
[min/mM]

	**$C_{0.00}$**			**$C_{0.04}$**			**$C_{0.05}$**		
	Mean	SD	%RSD	Mean	SD	%RSD	Mean	SD	%RSD
	6.91	0.08	1.20	8.62	0.12	1.43	10.53	0.14	1.39
	4.35	0.06	1.52	5.40	0.09	1.69	6.24	0.11	1.72
	3.04	0.05	1.78	3.62	0.07	1.92	4.59	0.10	2.25
	2.35	0.06	2.46	2.89	0.07	2.43	3.51	0.09	2.67
	2.01	0.06	2.77	2.62	0.07	2.51	3.12	0.09	2.92
	1.87	0.06	3.38	2.45	0.06	2.62	2.81	0.08	2.98
	1.79	0.06	3.42	2.29	0.06	2.58	2.71	0.08	3.06
	1.72	0.07	3.89	2.06	0.06	3.01	2.59	0.08	3.27
	1.68	0.07	3.92	1.95	0.07	3.37	2.38	0.09	3.77

Peak area for inhibitor (I) conentrations **$C_{0.00}$ - $C_{0.05}$**
[PA]

	Mean	SD	%RSD
	27490 - 239660	797.2 - 4074.2	1.7 – 2.9

Amount of the product
(p-nitrophenol) in the presence of inhibitor (I) concentrations **$C_{0.00}$ - $C_{0.05}$**
[mM/min]

	Mean	SD	%RSD
	0.029 - 0251	0.001 – 0.005	1.6 – 3.1

V_0 in the presence of inhibitor (I) concentrations **$C_{0.00}$ - $C_{0.05}$**
[mM/min]

	Mean	SD	%RSD
	0.09 – 0.60	0.001 – 0.024	1.22 – 3.92

$\frac{1}{Vo}$ in the presence of inhibitor (I) concentrations **$C_{0.00}$ - $C_{0.05}$**
[min/mM]

	Mean	SD	%RSD
	1.68 – 10.53	0.05 – 0.14	1.20 – 3.92
